# Effect of Prohydrojasmon on the Growth of Eggplant and Komatsuna

**DOI:** 10.3390/plants9101368

**Published:** 2020-10-15

**Authors:** Haidar Rafid Azis, Shinya Takahashi, Masami Koshiyama, Hiroshi Fujisawa, Hiroko Isoda

**Affiliations:** 1 Alliance for Research on the Mediterranean and North Africa (ARENA), University of Tsukuba, Tsukuba 305-8572, Japan; haidar.rafid.a@gmail.com (H.R.A.); isoda.hiroko.ga@u.tsukuba.ac.jp (H.I.); 2 Faculty of Life and Environmental Sciences, University of Tsukuba, Tsukuba 305-8572, Japan; 3 Specialty Chemical Division, Zeon Corporation, Chiyoda-ku, Tokyo 100-8246, Japan; M.Koshiyama@zeon.co.jp; 4 Headquarter, Zeon Corporation, Chiyoda-ku, Tokyo 100-8246, Japan; cepi@zeon.co.jp

**Keywords:** prohydrojasmon, inhibitory effect, plant hormone, komatsuna (*Brassica rapa* var. *periviridis*), eggplant (*Solanum melongena*)

## Abstract

Prohydrojasmon (PDJ) can improve the polyphenol and anthocyanin content and antioxidant activity of some crop plants, but it also shows a suppressive effect on the plant growth. This study aimed to investigate the inhibitory effect of PDJ on the growth of two crop plants: komatsuna (*Brassica rapa* var. *periviridis*) and eggplant (*Solanum melongena*). We applied various concentrations of PDJ drip-wise or by spraying to eggplant and komatsuna seedlings and made detailed observations of growth. In general, no significant suppressive effect of PDJ was observed in the aerial parts in both plants. However, a significant inhibitory effect was found in roots treated with PDJ at concentrations of 600 and 1000 ppm. Interestingly, komatsuna treated with PDJ at a concentration of 200 ppm in both approaches resulted in a significant increase in root weight up to 37%. At a concentration range of 200–400 ppm, PDJ showed no inhibitory effects, and in some cases slightly promoted root growth. Therefore, this could be the recommended concentration range. We conclude that application of PDJ can still be beneficial to the vegetable crops without causing serious inhibition or suppression effects on the growth, as long as it is kept at rather low concentrations.

## 1. Introduction

Jasmonates are among the most important plant hormones which play roles in many aspects of plant growth. As a class of oxylipins, jasmonates can induce a wide range of responses in higher plants. Since the first isolation of its methyl ester (jasmonic acid methyl ester), numerous jasmonates have been detected in various plant phyla. Jasmonates are found in algae, mosses, fungi, gymnosperms, and angiosperms [1,2,3].

Prohydrojasmon (PDJ) is a synthetic analog of jasmonic acid (JA) developed as a plant growth regulator. Plant growth regulators are defined as naturally or chemically synthesized substances that play roles in developmental or metabolic processes in plants [4]. The activity of PDJ may be similar to that of endogenous JA. It has been shown to affect several physiological processes, including senescence, leaf abscission, fruit ripening, coloration, and pigment accumulation [5,6]. Other studies have shown that PDJ functions as an elicitor in plants to stimulate the accumulation of secondary metabolites including phenolics, anthocyanins, terpenoids, and glucosinolates [7,8].

Numerous studies have reported that application of PDJ, like methyl jasmonate (MJ), can improve several aspects of crop quality. Application of PDJ has been reported to improve the hand-picking efficiency of satsuma mandarin [9]. PDJ applied to *Gala* and *Braeburn* apples a couple of days before harvest resulted in a significant increase in red color index [10]. Similarly, the red-blushed color in some cultivars of mangoes significantly improved after being treated with PDJ at low concentration a few weeks prior to harvest [11]. This kind of color development was also reported to have occurred in red pear after being sprayed with PDJ during the pre-color-change period [12]. The color improvements in those fruits are strongly associated with the accumulation of anthocyanins which is enhanced by the application of PDJ.

To date, we found that studies using PDJ majorly are still dominated by its application to fruit crops. In our previous study, however, we reported that application of PDJ to vegetable crops significantly increased the phenolic compounds, anthocyanin content, and antioxidant activity in komatsuna and lettuce [13].

Despite improvements of those secondary metabolites, PDJ is also known to have some kind of suppressive or inhibition effects on plant growth, particularly in initial plant growth [14]. In fact, growth inhibition was among the first characterized physiological effects of jasmonates, particularly root growth inhibition [15,16]. Previous studies have found that exogenous application of JA can inhibit various aspects of seedling growth including primary root growth, leaf expansion, and hypocotyl elongation [17,18,19]. One study emphasized that root growth is more sensitive to MJ [20]. Although this suppressive effect of PDJ is not as strong as that of MJ [21], it could still interrupt the plant growth, particularly if it is applied at higher concentrations. To answer how far the suppressive effect of PDJ could interrupt the growth of plants, here we conducted a study aimed at investigating the effect of PDJ at various concentrations on the growth and development of komatsuna and eggplant seedlings.

## 2. Results

### 2.1. Effect of PDJ Applied Drip-Wise on Growth of Komatsuna

**Figure 1:**
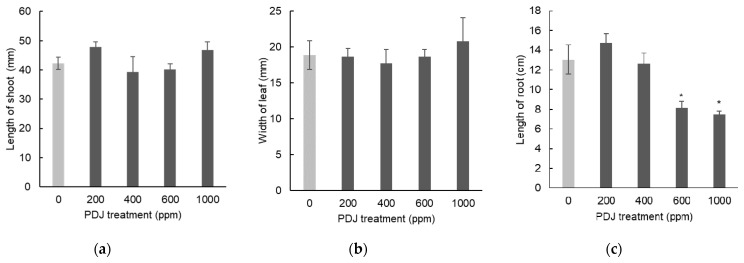
Shoot length (**a**), leaf width (**b**), and root length (**c**) of komatsuna seedlings treated with PDJ applied drip-wise at various concentrations. Komatsuna seeds were germinated and grown in soil, and PDJ was applied to seedlings at 1 week before harvest. Shoot length and leaf width values are the average difference between characteristics measured before and after treatment (*n* = 4). Root length is average value (*n* = 4) measured on the final day of pot culture (day 21). Error bars indicate standard error (SE). Asterisks (*) indicate statistically significant difference in comparison to the control using t-test at *p* < 0.05.

The application of PDJ drip-wise on komatsuna at the lowest and highest PDJ concentrations (200 and 1000 ppm, respectively) showed a tendency towards a slight increase in shoot length and its intermediate concentrations (400 and 600 ppm) showed an opposite tendency towards a slight decrease in shoot length. All results regarding this parameter were statistically insignificant (Figure 1a). The highest concentration of PDJ drip-wise (1000 ppm) showed a tendency towards a slight increase in leaf width, while the concentrations below that (200, 400, and 600 ppm) showed a tendency towards a slight decrease in leaf width. Similarly, all results regarding this parameter were statistically insignificant (Figure 1b). Interestingly, PDJ drip-wise at concentrations of 600 and 1000 ppm significantly reduced root length by 38% and 43%, respectively; the remaining concentrations (200 ppm and 400 ppm) showed statistically insignificant change in the root length (Figure 1c). PDJ applied by drip-wise approach seemingly did not show a distinctive change to the dimensional growth of the aerial part of komatsuna, however, at higher concentrations, the root length was significantly suppressed.

**Figure 2:**
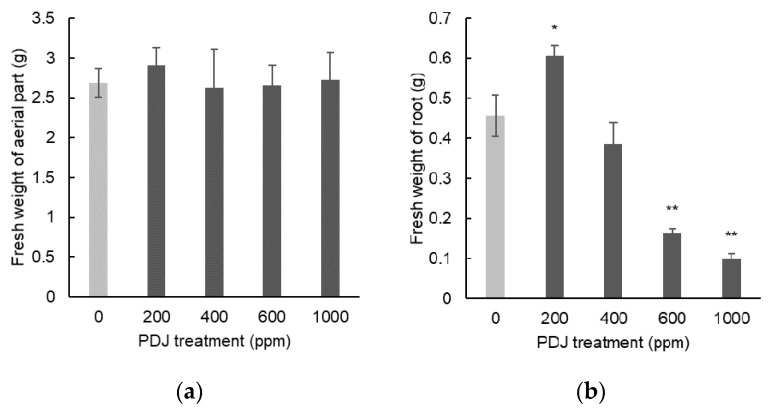
Weights of aerial parts (**a**) and roots (**b**) of komatsuna treated with PDJ applied drip-wise at various concentrations. Komatsuna seeds were germinated and grown in soil. PDJ at 200, 400, 600, and 1000 ppm was applied to seedlings at 1 week before harvest. Weight of aerial parts (**a**) and root weight (**b**) are average values (*n* = 4) measured on the final day of pot culture (day 21). Error bars indicate standard error (SE). Asterisks (* and **) indicate statistically significant difference in comparison to the control using t-test at *p* < 0.05 and *p* < 0.01, respectively.

The tendency towards an 8% and 2% increase in the weight of the aerial parts induced by 200 and 1000 ppm exposure levels of PDJ, respectively, was statistically insignificant, as well as the decline in these indices detected in the case of 400 and 600 ppm exposure levels (Figure 2a). In contrast, PDJ applied drip-wise at concentrations of 600 and 1000 ppm reduced the root weight by 66% and 79% (both *p* < 0.01), respectively, at 200 ppm showed a significant increase in the root weight by 26%, and at 400 ppm showed a slight decrease tendency in the root weight by 20%, relative to its control (Figure 2b). When it was applied drip-wise, especially at higher concentrations, PDJ highly reduced the weight of the root of komatsuna, and interestingly, stimulated the root growth at a concentration of 200 ppm. However, we did not observe significant change in the weight of the aerial parts, but merely an increase or decrease tendency.

### 2.2. Effect of PDJ Applied by Spraying on Komatsuna Growth

**Figure 3:**
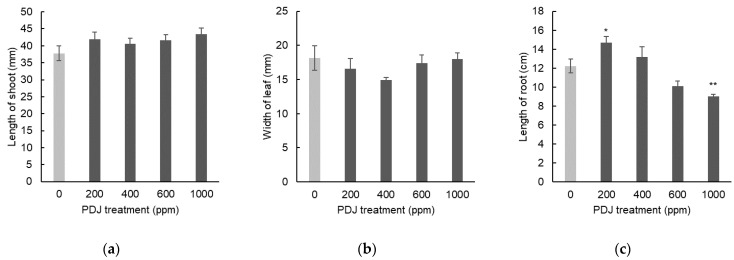
Shoot length (**a**), leaf width (**b**), and root length (**c**) of komatsuna seedlings sprayed with PDJ at various concentrations. Komatsuna seeds were germinated and grown in soil, and PDJ was sprayed onto seedlings at 1 week before harvest. Shoot length and leaf width values are the average difference between characteristics measured before and after treatment (*n* = 4). Root length is average value (*n* = 4) measured on the final day of pot culture (day 21). Error bars indicate standard error (SE). Asterisks (* and **) indicate statistically significant difference in comparison to the control using t-test at *p* < 0.05 and *p* < 0.01, respectively.

When PDJ was applied by spraying at concentrations of 200, 400, 600, and 1000 ppm, komatsuna showed a tendency towards a slight increase in shoot length by 11%, 8%, 10%, and 15%, respectively, relative to its control, which was statistically insignificant (Figure 3a). In contrast, PDJ sprayed at 200, 400, 600, and 1000 ppm showed a tendency towards a slight decrease in leaf width by 9%, 18%, 4%, and 1%, respectively, relative to its control, which was statistically insignificant as well (Figure 3b). Spray treatments with PDJ at 1000 ppm highly significantly inhibited root length by 26% (*p* < 0.01), at 600 ppm showed a decrease tendency in root length by 17% which was statistically insignificant, at 400 ppm showed an increase tendency in root length by 8% which was also statistically insignificant, and eventually at 200 ppm, it significantly increased root length by 20% (*p* < 0.05) (Figure 3c). Similar to the drip-wise approach, spray treatments with PDJ did not cause significant change to the dimensional growth of aerial parts of komatsuna, but again the higher concentration of PDJ significantly suppressed the root length of komatsuna, and interestingly, we found that at a concentration of 200 ppm, it also significantly increased the root length of komatsuna.

**Figure 4:**
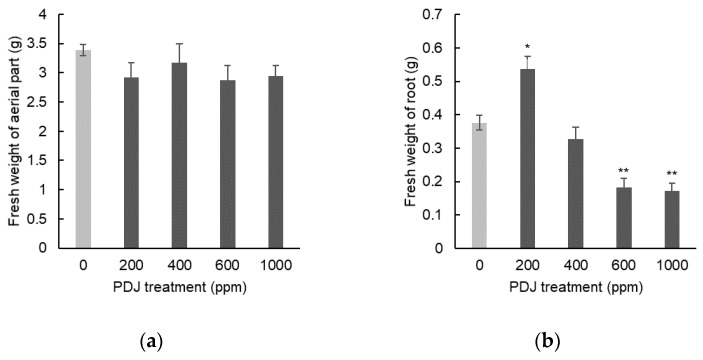
Weight of aerial parts (**a**) and roots (**b**) of komatsuna seedlings sprayed at various concentrations. Komatsuna seeds were germinated and grown in soil. Spray treatments of PDJ at 200, 400, 600, and 1000 ppm were applied to seedlings at 1 week before harvest. The weight of aerial parts (a) and roots (b) are shown as average values (*n* = 4) measured on the final day of pot culture (day 21). Error bars indicate standard error (SE). Asterisks (* and **) indicate statistically significant difference in comparison to the control using t-test at *p* < 0.05 and *p* < 0.01, respectively.

The tendency towards 14%, 6%, 15%, and 13% decrease in weight of aerial parts induced by 200, 400, 600, and 1000 ppm exposure levels of PDJ by spraying, respectively, compared to its control, was statistically insignificant (Figure 4a). Spraying with PDJ at concentrations of 600 and 1000 ppm reduced root weight by 52% and 54% (both *p* < 0.01), respectively; at 200 ppm, significantly increased root weight by 37%; and at 400 ppm, showed a tendency towards a decrease in root weight by 13%, relative to its control (Figure 4b). Regarding the weight data, the result of spraying was identical to the drip-wise approach, whereas the high concentration of PDJ highly reduced the root length of komatsuna, and the lowest concentration increased the root length significantly. Similarly, no significant changes were found in the weight of aerial parts of komatsuna treated by PDJ spray; every concentration only indicated a tendency towards increase or decrease.

**Figure 5:**
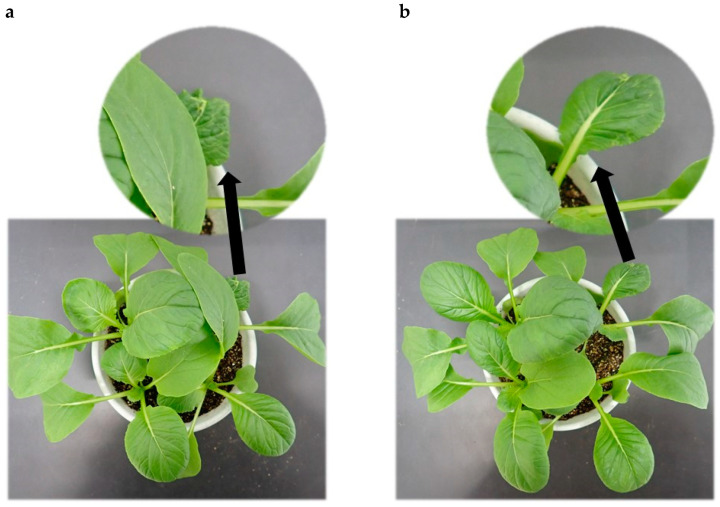
Wilting of komatsuna leaves. Pictures were taken at day 19 (**a**) and day 21 (**b**) and show wilting of komatsuna leaves after being sprayed with 1000 ppm PDJ.

**Figure 6:**
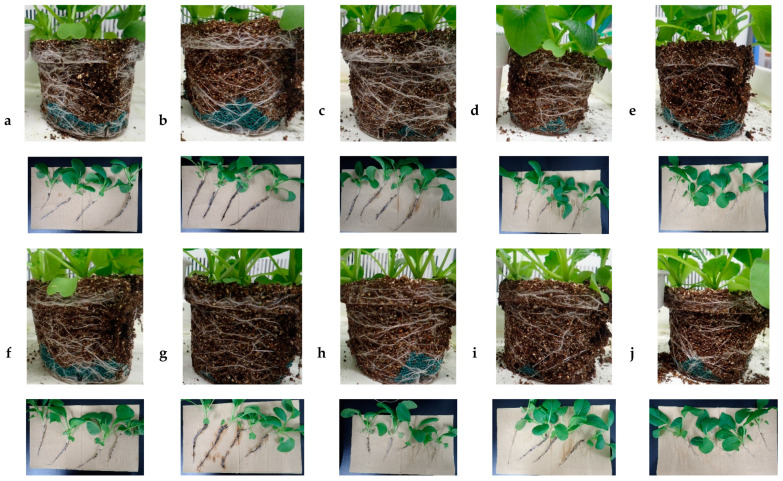
Effect of PDJ on root growth and development of komatsuna. Pictures were taken on the final day (day 21) of pot culture of seedlings treated with 0 (**a**,**f**), 200 (**b**,**g**), 400 (**c**,**h**), 600 (**d**,**i**), and 1000 (**e**,**j**) ppm PDJ applied drip-wise (**a**–**e**) or by spraying (**f**–**j**). PDJ at concentrations of 200 and 400 ppm (**b**,**g** and **c**,**h**) resulted in more complex, thicker, and stronger root networks, and at concentrations of 600 and 1000 ppm (**d**,**i** and **e**,**j**) resulted in less complex, thinner, and weaker root networks. In general, lower concentrations (200–400 ppm) of PDJ applied drip-wise, rather than by spraying, stimulated root growth and development. PDJ applied drip-wise led to stronger and more robust roots, and better incorporation between soil and roots; PDJ applied by spraying led to weaker and more fragile roots, and a crumbly and brittle (loose) soil structure.

At the highest concentration (1000 ppm), PDJ caused wilting/shrinking symptoms in some leaves of komatsuna on day 19 and day 21 of the pot culture (Figure 5). These symptoms may have been related to the onset of senescence. Strong responses to PDJ in terms of growth and development were observed in the root. Visually, at lower concentrations (200–400 ppm), PDJ led to the formation of a robust root network, while at higher concentrations (600–1000 ppm), the suppressive effect of PDJ resulted in a somehow frail and fragile root network (Figure 6).

### 2.3. Effect of PDJ Applied Drip-Wise on Growth of Eggplant

**Figure 7:**
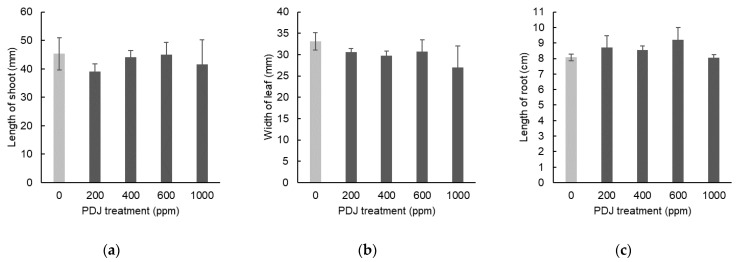
Shoot length (**a**), leaf width (**b**), and root length (**c**) of eggplant seedlings treated with PDJ applied drip-wise at various concentrations. Eggplant seeds were germinated and grown in soil. PDJ was applied to seedlings at 1 week before harvest. Shoot length and leaf width values are the average difference between characteristics measured before and after treatment (*n* = 4). Root length is the average value (*n* = 4) measured on the final day of pot culture (day 28). Error bars indicate standard error (SE).

In eggplant, PDJ applied drip-wise at concentrations of 200, 400, 600, and 1000 ppm showed a tendency towards a slight decrease in shoot length by 14%, 3%, 1%, and 8%, respectively, relative to its control, which was statistically insignificant (Figure 7a). Similarly, PDJ applied drip-wise at concentrations of 200, 400, 600, and 1000 ppm showed a tendency towards a slight decrease in leaf width by 8%, 10%, 7%, and 19%, respectively, relative to its control, which was insignificant as well (Figure 7b). In terms of root length, PDJ applied drip-wise at concentrations of 200, 400, and 600 ppm showed a tendency towards a slight increase in root length by 8%, 6%, and 14%, respectively, and at 1000 ppm, showed a decrease tendency in root length by 0.3% relative to its control, which was statistically insignificant (Figure 7c). Since there was no significant change observed within all of these data, it seems that the dimensional growth of eggplant was not affected by the PDJ through the drip-wise approach. All decrease or increase cases showed merely a tendency rather than stimulating or suppressing the growth by PDJ.

**Figure 8:**
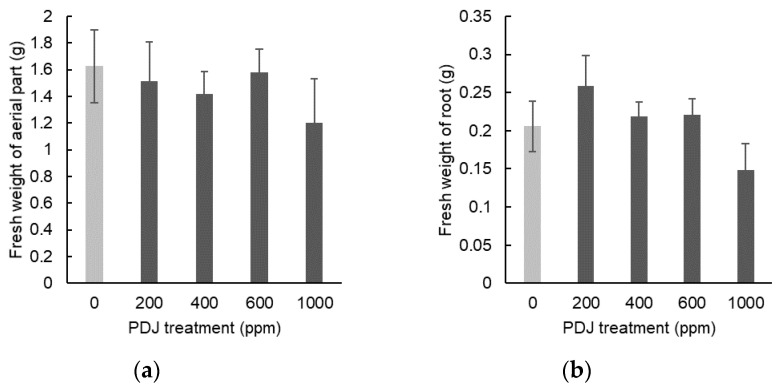
Weight of aerial parts (**a**) and roots (**b**) of eggplant seedlings treated with PDJ applied drip-wise at various concentrations. Eggplant seeds were germinated and grown in soil. PDJ was applied drip-wise at concentrations of 200, 400, 600, and 1000 ppm to seedlings at 1 week before harvest. Weight of aerial parts (a) and roots (b) are shown as the average value (*n* = 4) measured on the final day of pot culture (day 28). Error bars indicate standard error (SE).

When applied drip-wise at concentrations of 200, 400, 600, and 1000 ppm, PDJ showed a tendency towards a slight decrease in the weight of the aerial parts by 7%, 13%, 3%, and 26%, respectively, relative to its control, which was statistically insignificant (Figure 8a). In contrast, PDJ applied drip-wise at concentrations of 200, 400, and 600 ppm showed a tendency towards a slight increase in root weight by 26%, 6%, and 7%, respectively, and at 1000 ppm showed a decrease tendency in root weight by 28%, relative to its control, which was also insignificant statistically (Figure 8b).

Unlike in komatsuna, PDJ applied drip-wise did not show significant change in the weight of eggplant, either in the aerial parts or in the root part. However, we observed that the highest concentration of PDJ showed a strong tendency towards weight reduction in the root, yet statistically insignificant. An identical pattern was found for PDJ at 200 ppm showing tendency towards increase in particular root weight, but then again it was not significant.

### 2.4. Effect of PDJ Applied by Spraying on Growth of Eggplant

**Figure 9:**
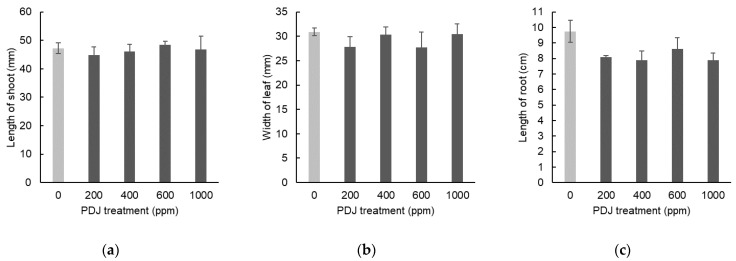
Shoot length (**a**), leaf width (**b**), and root length (**c**) of eggplant seedlings sprayed with PDJ at various concentrations. Eggplant seeds were germinated and grown in soil, and PDJ was applied to the seedlings by spraying at 1 week before harvest. Shoot length and leaf width values are the average difference between characteristics measured before and after treatment (*n* = 4). Root length is average value (*n* = 4) measured on the final day of pot culture (day 28). Error bar indicates standard error (SE).

In eggplant, PDJ sprayed at concentrations of 200, 400, 600, and 1000 ppm showed a tendency towards a slight decrease in shoot length by 5%, 2%, 3%, and 1%, respectively, which was statistically insignificant relative to its control (Figure 9a). Similarly, PDJ applied by spraying at concentrations of 200, 400, 600, and 1000 ppm showed a tendency towards a slight decrease in leaf width by 10%, 2%, 10%, and 1%, respectively, which was also insignificant relative to its control (Figure 9b). Application of PDJ by spraying at concentrations of 200, 400, 600, and 1000 ppm also showed a tendency towards a slight decrease in root length by 17%, 19%, 12%, and 19%, respectively, which was insignificant as well relative to its control (Figure 9c). These differences were not statistically significant but reflected a trend of PDJ applied by spraying that tends to suppress the dimensional growth of eggplant seedlings.

**Figure 10:**
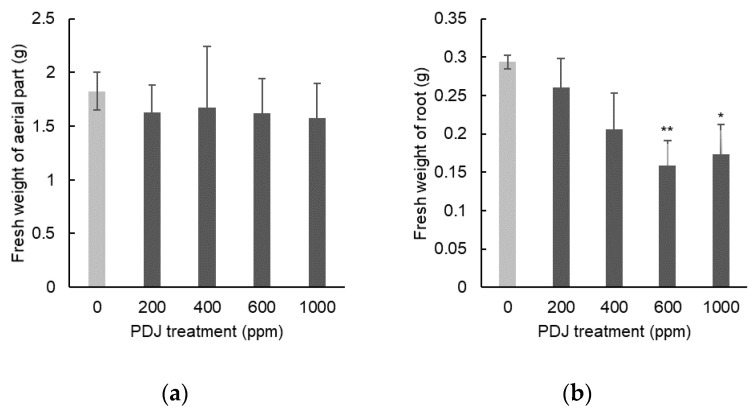
Weight of aerial parts (**a**) and roots (**b**) of eggplant seedlings sprayed with PDJ at various concentrations. Eggplants were germinated and grown in soil. Spray treatments of PDJ at 200, 400, 600, and 1000 ppm were applied to the seedlings at 1 week before harvest. Weights of aerial parts (**a**) and roots (**b**) are shown as average values (*n* = 4) measured on the final day of pot culture (day 28). Error bar indicates standard error (SE). Asterisks (* and **) indicate statistically significant difference in comparison to the control using t-test at *p* < 0.05 and *p* < 0.01, respectively.

Spray applications of PDJ to eggplant at concentrations of 200, 400, 600, and 1000 ppm showed a tendency towards a slight decrease in weight of aerial parts by 11%, 8%, 11%, and 14%, respectively, which was statistically insignificant relative to its control (Figure 10a). PDJ sprayed at a concentration of 1000 ppm reduced root weight significantly by 41%; at a concentration of 600 ppm reduced root weight highly significantly by 46%; and at a concentration 400 and 200 ppm only showed a tendency towards a slight decrease in root weight by 11% and 30%, respectively, which was statistically insignificant relative to its control (Figure 10b).

The result regarding weight of aerial parts in eggplant by PDJ spray was similar to that by PDJ drip-wise, in which generally they showed a decrease tendency. However, regarding root weight, PDJ spray at higher concentrations showed a significant reduction, yet the concentration of 200 ppm did not show an increase tendency like that observed in PDJ drip-wise, but rather a decrease tendency.

**Figure 11:**
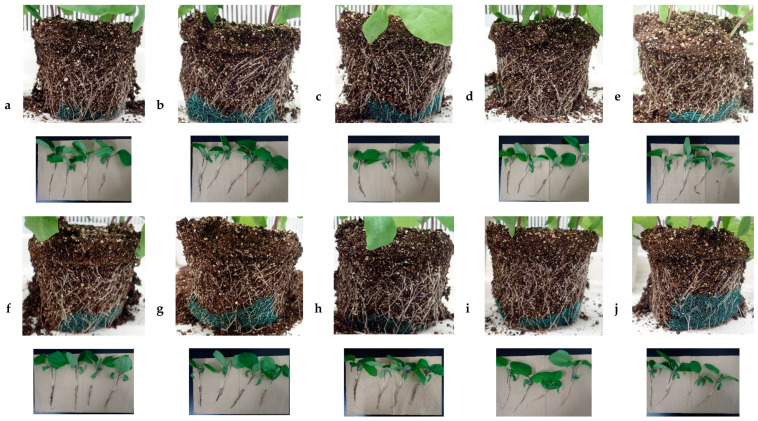
Effect of PDJ on eggplant root growth and development. Pictures were taken on the final day (day 28) of pot culture of seedlings treated with 0 (**a** and **f**), 200 (**b** and **g**), 400 (**c** and **h**), 600 (**d** and **i**), and 1000 (**e** and **j**) ppm PDJ supplied drip-wise (**a**–**e**) or by spraying (**f**–**j**). PDJ at concentrations of 200 and 400 ppm (**b**,**g** and **c**,**h**) resulted in more complex, thicker, and stronger root networks, while PDJ at concentrations of 600 and 1000 ppm (**d**,**i** and **e**,**j**) resulted in less complex, thinner, and weaker root networks. In general, lower concentrations (200–400 ppm) of PDJ applied drip-wise, rather than by spraying, stimulated root growth and development. Drip-wise application of PDJ led to stronger and more robust roots, and better incorporation between soil and roots, while spraying of PDJ led to weaker, fragile roots and a crumbly and brittle (loose) soil structure.

Similar to komatsuna, no significant result was shown in dimensional growth of aerial parts in eggplant. While PDJ drip-wise did not show any significant change in all growth parameters, PDJ spray at higher concentrations reduced the root weight of eggplant significantly, similar to the komatsuna case. Regarding the root, visually, it seems that lower concentrations (200–400 ppm) of PDJ led to the formation of robust, dense, and complex root networks, while the higher concentrations (600–1000 ppm) resulted in a somehow frail and fragile root network due to the distinct suppressive effect of PDJ, which was practically identical in komatsuna (Figure 11).

## 3. Discussion

We found that PDJ indeed had an effect on the growth of plants, whether it stimulated or suppressed the growth in both approaches. This effect, however, was mild, especially in terms of dimensional growth (length of shoot and width of leaves) and the weight of aerial parts. PDJ at low concentration showed a stimulatory tendency towards growth, while at high concentration showed the opposite effect of inhibition tendency. In most cases, a higher concentration resulted in significant growth suppression of the root development, particularly root weight. However, in the aerial part, we found a wilting leaves phenomenon occurred in komatsuna after they sprayed with PDJ at a high concentration (1000 ppm). This phenomenon might be associated with senescence.

Senescence in plants can be defined as the degeneration process of cells, organs, or the entire organism, leading to death [22]. It has been reported that treatment with MJ can lead to plant senescence. Because MJ is volatile and odoriferous, it can be directly incorporated into the internal leaf tissues during gas exchange, and then eventually promote senescence [23]. Since PDJ is an analog of MJ [10], we expect that PDJ may have a similar effect to induce senescence.

Plants that are strongly negatively affected by PDJ (especially at high concentration) might show wilting/shrinking symptoms, like in the komatsuna case of this study. However, senescence is a highly complex process controlled by multiple layers of regulation and it is an integral part of the plant lifecycle. Furthermore, senescence is not a single state, and should be understood from a temporally dynamic perspective [24]. Thus, it requires further analysis to reveal whether this wilting phenomenon is directly caused by senescence or not. 

Previous studies have shown that aerial plant parts, especially leaves, are affected by JA. For example, a study on *Arabidopsis thaliana* showed that JA application inhibited the expansion of true leaves and cotyledons. In addition, JA was shown to inhibit the activity of mitotic cyclin CycB1;2, which, in turn, repressed leaf expansion. It has been reported that the *Arabidopsis* COI1-JAZ-MYC2 cascade mediates the JA-induced inhibition of leaf expansion [25]. However, it seems that PDJ has less or weaker inhibition effect compared with JA, particularly as shown by the results of this study with respect to all dimensional growth parameters in komatsuna and eggplant as well. Mostly, none of these data showed a significant result, with the exception of the length of root results in komatsuna.

**Figure 12:**
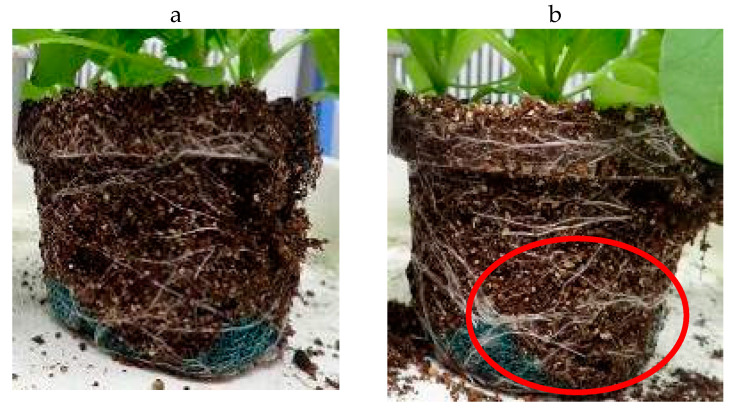
PDJ drip-wise (**a**) showed stronger effect than PDJ spray (**b**). Both pictures were taken on the final day of pot culture (day 28). The left picture shows a smaller amount of roots and thinner roots in komatsuna treated by PDJ drip-wise at a concentration of 1000 ppm. The right picture shows relatively more remaining roots (indicated by red circle) and thicker roots in komatsuna treated by PDJ spray at a concentration of 1000 ppm.

In terms of root development, we observed that at higher concentrations (600–1000 ppm), PDJ applied drip-wise tended to show a stronger suppression effect than PDJ applied by spraying. Figure 12 shows that high concentrations of PDJ applied drip-wise resulted in less dense and a smaller amount of roots than PDJ applied by spraying. This might have happened due to the direct exposure of PDJ drip-wise, which directly targets the soil. However, interestingly, at lower concentrations (200–400 ppm), drip-wise application of PDJ tended to result in rather stronger, thicker, and more complex root networks, and caused the soil to become more compact and packed. Meanwhile, PDJ applied by spraying in general tended to result in rather fragile, thinner, and less complex root networks, and a crumbly and brittle soil structure. In addition, application of PDJ by spraying resulted in a rather crumbly and brittle soil structure, whereas the drip-wise application of PDJ tended to result in more compact and packed soil.

Application of JA and its derivatives may have different effects on plants, depending on the species. In general, JA affects the formation of lateral and adventitious roots [17]. One study reported that JA upregulated the expression of *ERF109*, which promoted lateral root formation in *Arabidopsis* [26,27]. Another study found that JA enhanced adventitious root formation in petunia cuttings [28].

The changes in root structure in eggplant were similar to those in komatsuna, but the root system of eggplant was much larger than that of komatsuna. Changes in the root parts might be associated with changes in cell proliferation and cell elongation caused by PDJ [29]. Another study reported that growth inhibition is associated with the inhibition of oxygen and water uptake during germination and seedling growth caused by JA [20].

Our results show that the tendency of inhibition caused by PDJ was mild in lower conditions, and it was milder in eggplant than in komatsuna. In another perspective, it can be seen that eggplant is somehow stronger in dealing with this so-called suppressive effect of PDJ. This might be due to differences in the maturity level between these two crops. It may also be related to the high phenolics content in eggplant.

Because of its phenolic constituents, eggplant is known as one of the top 10 vegetables in terms of its oxygen radical scavenging capacity [30]. A study on the distribution of total phenolic contents in eggplant reported high concentrations of polyphenols in the leaves and stems, as well as in the fruit [31]. Phenolics are found abundantly in plant tissues and are actively involved in defense against biotic and abiotic stresses [32]. Therefore, the presence of phenolics in eggplant may indicate a high level of resistance against stresses. In this study, the stress factor practically was the PDJ exposure.

Eggplant, particularly that treated by PDJ spraying, did not show significant inhibitory effects in all growth parameters. Somehow it is stronger and more resistant than komatsuna when dealing with the exposure to PDJ which was expected to have an inhibition effect. However, to some extent, we still detected a trend of suppression tendency caused by PDJ, whether applied drip-wise or by spraying, yet in most cases, this effect was not significant.

Among all the data of dimensional growth and weight of plant aerial parts, none of them showed significant results. Therefore, the decrease or increase that occurred can only be seen as a tendency. However, it is a good sign knowing that PDJ actually did not show a serious suppressive effect on the aerial parts of plants in general. The wilting/shrinking phenomenon that was found in komatsuna occurred only for PDJ applied as a spray while it was not found for PDJ applied drip-wise and in eggplant in any approaches used. We presumed that the inhibition effect of PDJ applied by spraying can only last for about 3–4 days and afterward this effect slowly subsides. However, at high concentrations, this inhibition effect is seemingly much stronger and eventually causes wilting leaves in komatsuna regardless of the subsided effect after four days have passed.

## 4. Materials and Methods 

### 4.1. Plant Materials, Growth Conditions, and PDJ Treatments 

Komatsuna (*Brassica rapa* var. *periviridis*) and eggplant (*Solanum melongena*) seeds were purchased from the Takaii Seed Co. (Kyoto, Japan). Four seeds of each plant were sown directly into each pot (diameter = 10.5 cm and depth = 9 cm) containing soil (Metro Mix, HYPONeX Japan Co., Osaka, Japan) moistened with tap water. Five pots were used: one for each treatment, and a control treated with distilled water. The potted plants were kept in a growth chamber (LPH-214-S, NK system, Japan).

The growth conditions were set to 23 °C and ca. 60% relative humidity with a 14 h light/10 h dark photoperiod. Light was supplied by white fluorescent lights (FHF-16EX-N-H, NEC Lighting Ltd., Tokyo, Japan). The photosynthetic photon flux density (PPFD) was ca. 110 μmol m^−2^ s^−1^ at the top of each plantlet. Distilled water was added to all the pots evenly and regularly to maintain soil moisture. To support growth, additional fertilizer (Hyponex N-P-K = 6-5-10, HYPONeX Japan Co., Osaka, Japan), diluted 1:500, was supplied to all the pots once per week after the first true leaves had completely emerged.

Jasmomeito^®^ Ekizai (Meiji-Seika Pharma Co., Yokohama, Japan), which contains 5% of the active ingredient PDJ, was prepared at 200, 400, 600, and 1000 ppm concentrations by dilution with distilled water. These PDJ treatments were applied by two approaches. The first one was by the foliar spraying approach in which the PDJ was sprayed towards the aerial parts of the plant. The second approach was by the drip-wise approach using a pipet in which PDJ was dripped directly into the soil. The volume of PDJ applied was 1 mL in both approaches and crop plants. PDJ was applied one week prior to harvest. Distilled water served as the control. The duration of pot culture for komatsuna and eggplant was 21 days and 28 days, respectively.

### 4.2. Plant Growth Measurements

Plant growth was carefully observed and measured on the last week of the pot culture. Shoot length and leaf width were measured one day prior to treatment and then every two days until the final day. In this experiment, shoot length refers to the length of epicotyl of the longest first true leaves, measured from the base of true leaves to the tip. Leaf width was measured at the widest part of the largest true leaf. All measurements of dimensional growth were conducted using calipers.

On the final day of pot culture, all plants were collected. The roots were washed with water to remove soil. Then, the plants were cut into two parts: aerial parts and roots. The root length, root fresh weight, and fresh weight of aerial parts were measured, recorded, and further analyzed. All data presented are average values (*n* = 4).

### 4.3. Statistical Analysis 

All statistical analyses were conducted using Microsoft Excel. Student’s t-test was used to assess statistical differences between the assay results of PDJ treatments and controls. A value of *p* < 0.05 was considered significant (*) and *p* < 0.01 highly significant (**).

## 5. Conclusions

In conclusion, our findings indicated that PDJ did not show a significant inhibitory effect on the growth of komatsuna and eggplant. The suppression or stimulation effect by PDJ was merely a tendency, particularly in dimensional growth as well as weight of aerial parts. Application of PDJ, therefore, can still be beneficial to improve the quality of crop plants without causing the inhibition issue to the growth of the plants. However, we noticed that special attention needs to be taken when using PDJ at rather higher concentrations, in this case, PDJ at concentrations of 600 ppm and 1000 ppm. Based on our findings, PDJ used within this concentration range may start to significantly cause adverse effects on the growth of plants, particularly on the root growth, indicated by a drastic reduction of root weight (with the exclusion of spray application to eggplant). As long as the concentration of PDJ is kept low, we expect that these kinds of inhibition effects of PDJ will not affect the growth, and probably will only show a tendency towards a slight inhibition. In contrast, there are some cases showing a tendency and even significant stimulating effect on the growth of plants when PDJ at low concentration (200 ppm) is applied. Therefore, we suggest that it is much safer and better to use PDJ at low concentrations and based on this study, we propose PDJ in the concentration range of 200–400 ppm as the recommended concentration.
